# Sleep, Behavior, and Adaptive Function in KAT6A Syndrome

**DOI:** 10.3390/brainsci11080966

**Published:** 2021-07-23

**Authors:** Clay Smith, Jacqueline Harris

**Affiliations:** Kennedy Krieger Institute, Baltimore, MD 21205, USA; smithcl@kennedykrieger.org

**Keywords:** KAT6A, epigenetics, sleep, behavior

## Abstract

KAT6A syndrome is a Mendelian Disorder of the Epigenetic Machinery characterized by intellectual disability and profound expressive language impairment. This study aimed to further characterize behavior and sleep in this syndrome. 26 participants between the ages of 3 and 35 years with KAT6A syndrome were assessed via parental informant using the Adaptive Behavior Assessment System version 3 (ABAS-3), Achenbach Child or Adult Behavior Checklist (CBCL/ABCL), and a Modified Simonds and Parraga Sleep Questionnaire (MSPSQ). The ABAS reports conceptual, social, and practical domains of adaptive function as well as a general composite score for adaptive function. The CBCL/ABCL is an inventory that measures internalizing, externalizing, and DSM-oriented problem domains. The MSPSQ is a mix of qualitative and quantitative sleep information that includes behavioral and medical sleep problems. Mean values for all domains of the ABAS-3 were in the extremely low range. Additionally, sleep was very dysfunctional in this cohort. Sixty percent of respondents reported feeling there was a sleep problem, 64% take medication for sleep, and 68% have sought treatment or advice for sleep. Only 12% of these participants have sleep apnea suggesting that sleep problems in this disorder are unrelated to sleep-disordered breathing. Interestingly, there were extremely low rates of all types of behaviors reported among participants on the CBCL/ABCL. No significant differences were seen based on genotype grouping in adaptive function, sleep, or behavior. This study further delineates the phenotype of the KAT6A syndrome and emphasizes the need for supports for adaptive functioning as well as detailed attention to the behavioral aspects of sleep in this condition.

## 1. Introduction

KAT6A syndrome (Arboleda–Tham syndrome; ARTHS; MIM#616268) is a genetic disorder first characterized in 2015 [1,2,3]. These initial descriptions included 10 affected individuals and to date, there are just over 80 cases published in the literature. Some phenotypic variability exists among reported cases though developmental delay/intellectual disability is a universal feature. Other notable phenotypic features include microcephaly, neonatal hypotonia, reflux, constipation, general feeding difficulties, congenital heart defects, behavioral problems, and sleep disturbance [4].

KAT6A serves to acetylate lysine-9 on histone 3 (H3K9) and functions as a transcriptional coactivator [5]. Genetic variants that impair these functions lead to downstream dysregulation of other vital proteins. For example, murine models with disrupted KAT6A histone acetyltransferase (HAT) function led to early replicative senescence in hematopoietic and neural progenitor cells through dysregulation of p16 [6]. To date, most pathogenic variants found in humans have been a protein-truncating loss of function variants. The majority reported are in the last two exons—exons 16 and 17 [4]. Syndromes caused by genetic variants that disrupt epigenetic modifiers like KAT6A are known as Mendelian disorders of epigenetic machinery (MDEMs). MDEMs are overrepresented in genetic etiologies of intellectual disability [7].

There are several examples of preclinical studies showing the post-natal rescue of neurological and functional outcomes in MDEMs [8,9,10]. Any future clinical outcome measures will require specific and refined neurologic and behavioral phenotypes. To date, information about the specific neurobehavioral phenotype of KAT6A syndrome is limited. Clinician-rated severity of intellectual disability shows a bias towards individuals with late-truncating variants being more severely affected and those with early-truncating variants having mild ID [4]. Speech delay, too, is universal, and reports also note profound impairment with the suggestion that it may be disproportionate to cognitive and receptive abilities. Little beyond this is known. We administered a set of parent-reported questionnaires to further understand behavior, adaptive function, and sleep in individuals with KAT6A syndrome. Here we present a general profile of very low adaptive function, significant sleep problems, and unexpectedly low rates of problematic behavior.

## 2. Materials and Methods

We recruited 26 individuals with a molecular diagnosis of KAT6A syndrome from our Epigenetics and Chromatin Clinic (https://www.hopkinsmedicine.org/institute-genetic-medicine/patient-care/genetics-clinics/epigenetics-chromatin-clinic/ accessed on 22 July 2021) or through appeals to the KAT6A foundation. Individuals aged 3 years and older were included at the time of recruitment. Individuals with primary languages other than English were not included. See supplementary materials for individual participant and variant data (Table S1). The study was approved by the Johns Hopkins Medical Institutions Institutional Review Board (NA_00079185), and all participants underwent a written informed consent process.

Age-appropriate versions of Child Behavior Checklist (CBCL) or Adult Behavior Checklist (ABCL), Adaptive Behavior Assessment System-Third Edition (ABAS-3), and Modified Simonds & Parraga Sleep Questionnaire (MSPSQ) were distributed to parents for reporting.

Behavior checklists from the Achenbach System of Empirically Based Assessment were utilized for assessing behavioral and emotional problems. The CBCL/1 ½-5, CBCL/6-18, and ABCL were used. Responses are given via Likert-scale and reports are normed by gender and age band with those beyond the 97th percentile being clinically significant. Each age-based version reports scales for internalizing, externalizing, and total problems as well as DSM-oriented scales for depressive, anxiety, and attention-deficit/hyperactivity problems [11,12,13].

The Adaptive Behavior Assessment System, third edition (ABAS-3) is a comprehensive measure built upon the ABAS-2 with goals of improving accuracy in assessments of those with intellectual disabilities [14]. Comparison studies of the ABAS, 2nd and 3rd editions have shown they are well correlated though with an increase in scores in the latter version [14,15]. The ABAS-3 generates standard scores with a mean of 100 and a standard deviation of 15 in conceptual, social, and practical domains with a general adaptive composite score. The ABAS-3 reports on 10 adaptive skill areas. Conceptual, social, and practical adaptive domains as well as a general adaptive composite (GAC) are reported in standard scores and percentiles. The 10 skill areas reported include communication, community use, functional academics, home living, health and safety, leisure, self-care, self-direction, social, and work. A standard score of 100 is average with a standard deviation of 15.

Wiggs and colleagues expanded the Simonds and Parraga Sleep Questionnaire for use in individuals with intellectual disabilities by adding some multiple-choice questions and responses as well as expanding open-ended questions for more qualitative information [16,17]. The questionnaire consists of more than 50 questions focusing on the previous month. The first section pertains to sleep environment, routine, and quality. Responses are generally open-ended with some duration and frequency reporting based on a 5–6 item Likert-type scale (few minutes, up to half an hour, up to one hour, between 1–2 h, more than 2 h and never, about once a month, a few times a month, one to two times a week, many times a week, daily). The second section consists of 30 questions pertaining to parasomnias, initiation and maintenance, sleep-disordered breathing, and daytime symptoms such as drowsiness and hyperactivity. Response choices are the Likert-type selections mentioned above. The final block consists of questions about any treatment received, primary and secondary sleep problems in the family, and whether they would consider their child to have a sleep problem. The Modified Simonds and Parraga Sleep Questionnaire (MSPSQ) was chosen for its expansiveness in questions and responses given the exploratory nature of this study. A convention for scoring this questionnaire by assigning scores to the quantitative responses has been developed and utilized previously [18,19,20,21]. However, we report here only the frequency of select individual responses based on our clinical judgment. Prolonged sleep initiation was defined as taking an hour or more to fall asleep at night. Problems with sleep maintenance were defined as waking up more than once per night or staying awake for longer than an hour. Bruxism, snoring, and needing medication were reported if occurring monthly or more. Restless sleep was defined as moving around a lot in bed a few times per month or more. Any episodes of apnea over the month were considered positive. Enuresis and daytime drowsiness were restricted to those 7 years and older to exclude those where it may be developmentally appropriate. The former was reported if happening monthly or more. The latter was defined as drowsiness or an irresistible urge to sleep occurring multiple times per month. Questions regarding treatment received or considering their child to have a sleep problem were dichotomous. Individuals submitted original reports of genetic variant information from performing labs. Original reports, documentation from treating clinicians, ClinVar, and Mutation Taster were utilized in assessing pathogenicity [22,23].

## 3. Results

### 3.1. Participants and Molecular Testing

**Figure 1:**
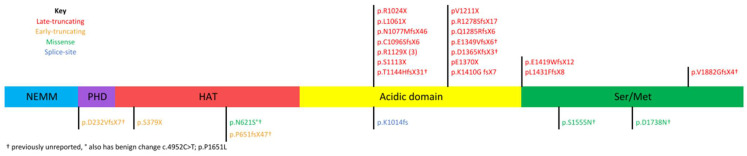
KAT6A domains and distribution of participants’ variants. NEMM, N-terminal part of Enok, MOZ, or MORF; PHD, plant homeodomain; HAT, histone acetyltransferase; Ser/Met, serine/methionine-rich region.

Twenty-six individuals with KAT6A syndrome were included in the study. Their ages ranged from 3 to 35 years of age (11.3 ± 8.9). One-half, 13/26, were male. Nine variants in this group have not previously been published. Nineteen individuals possess late-truncating frameshift (four newly reported here) or nonsense variants. Three individuals have early-truncating variants and 2 have frameshift mutations resulting in early truncation—both of these have not been reported. Three individuals possess novel missense variants reported here for the first time that are classified as likely pathogenic and 1 has a previously reported splice-site variant (Figure 1).

### 3.2. Adaptive Function

The adaptive function was almost universally impaired without any prevailing strengths evident from one domain to the other (Table 1). Twenty of 24 individuals had at least one measure < 2 standard deviations (SD) below the mean. Twenty-three of 24 individuals had at least one measure < 1.5 SD below the mean. A single individual had outlying numbers with standard scores in the 90s for each domain and the composite score.

Conceptual domain standard scores ranged from 44–95 (61 ± 11.7). Scores were very similar between those with late-truncating, early-truncating, and missense/splice-site variants. Social domain standard scores ranged from 53–99 (67.2 ± 12.2). No significant difference existed between variant groups though early truncating and missense/splice-site groups tended to higher scores. Practical domain standard scores ranged from 48–94 (60.9 ± 13.1). No significant difference was found between variant groups through missense/splice-site had a higher average with a large SD. GAC standard scores ranged from 44–95 (60.1 ± 12.8) with no significant difference between variant groups. Again, a modest trend towards higher scores for the missense/splice-site group though again with a large SD.

With 24 respondents, 3 domains, and a GAC—96 standard scores were reported. 74% of those were ≤2 SD below the mean. Six individuals accounted for 92% of the standard scores > 2 SD below the mean. There was no correlation between age and any domain of the adaptive function.

### 3.3. Behavior

In general, there were much fewer individual scores reaching clinical significance in the behavior measures as compared to the adaptive measures (Table 1). With 22 respondents and 6 problem areas—only 24 of a possible 132 problem areas (18%) were clinically significant. The T scores for internalizing problems ranged from 34–68 (54.8 ± 9). T scores for externalizing problems ranged from 33–70 (51.6 ± 10.2). Those for total problems ranged from 38–71 (58 ± 9.7). DSM-oriented scales for depressive, anxiety, and attention-deficit/hyperactivity problems yielded similar results. T scores for depressive problems ranged from 50–72 (60.6 ± 7.8), anxiety problems ranged from 50–72 (55.6 ± 6.3), and ADHD from 50–76 (60.3 ± 8.2). There was no correlation between age and any behavioral problems. Additionally, there was no correlation between the amount of behavioral problems and any domain of adaptive functioning.

### 3.4. Sleep

Sleep issues were very prominent among respondents (Table 2). More than 1/3 of respondents reported sleep latency of up to one hour or more. More than 1/2 were awakening multiple times per night or sleep interruptions were lasting an hour or more. More than half, 56%, experienced bruxism. Recurrent restless sleep plagued a majority, or 80%, of individuals. Snoring and apnea were not the predominant sleep problem but were still seen in a modest number of patients at 24% and 12% respectively. Apnea appeared to be more common in those with missense mutations but sample sizes were small. Rates of daytime somnolence/drowsiness and enuresis among those 7 years and older were both 72%. Interestingly, 60% reported perceived sleep problems though 64% reported needing medication for sleep and 68% have received advice or treatment for sleep issues. None of the sleep problems in the survey were correlated with lower adaptive functioning in any domain. There were no correlations between sleep problems and any behaviors with the exception of sleep-disordered breathing which was correlated with total behavior issues (r = 0.48, *p* = 0.03), internalizing (r = 0.45, *p* = 0.048), and externalizing behaviors (r = 0.56, *p* = 0.01) at a significant level.

## 4. Discussion

Previous studies have shown that low adaptive function and sleep problems independently predict behavioral problems in children and adults with disabilities [24,25]. However, respondents with KAT6A syndrome have remarkably low levels of maladaptive behaviors for any group of individuals with intellectual disability particularly a group with low adaptive abilities and problematic sleep [26]. Additionally, poor sleep does not appear to predict more difficult behaviors in this population with the exception of sleep-disordered breathing. This is not the case for many other genetic syndromes [27,28]. This may be because the overall rates of problematic behavior in this population are notably low. This behavioral phenotype needs to be further explored in both preclinical models and larger clinical populations with more direct assessment tools. Adaptive abilities were uniformly depressed without relative strengths and weaknesses among domains. The mean for all domains in these participants was >3 SD below the population mean. This suggests the need for special attention to accommodations and supports for adaptive functioning in patients with this syndrome.

Sleep issues among our patients are striking. More than ¾ have recurrent restless sleep. More than half require medication for sleep and/or have sought medical care for sleep. This prevalence of general sleep issues is more similar to disorders with a known disrupted sleep component such as Angelman syndrome or Rett syndrome than other genetic syndromes causing intellectual disability such as Fragile X or Down syndrome or Tuberous Sclerosis Complex [29]. Rates of snoring and apnea were similar to many other genetic syndromes such as Fragile X syndrome and Rett syndrome and lower than others such as Down syndrome and Prader-Willi syndrome [29]. Apnea and snoring only accounted for a small percentage of the overall group of sleep problems in this KAT6A population. Treating clinicians should thoroughly assess for sleep issues beyond sleep-disordered breathing in this population. Further characterization of sleep issues with actigraphy or polysomnography would be important next steps to better understand this sleep phenotype and guide treatment and potential outcome measures for research.

In addition to looking for general trends among all participants, this project sought to extend genotype-phenotype correlations proposed in Kennedy 2019. However, the low group numbers in the non-late truncating groups preclude any conclusions. Non-significant trends towards higher adaptive function and more sleep-disordered breathing in patients with missense/splice-site variants compared to the protein-truncating groups were seen. It would be consistent with trends seen in other MDEMs and with the previous research in KAT6A syndrome for those with missense variants to have higher cognitive functioning than those with truncating mutations.

Our study has several limitations. First, although it is a large sample size for this rare disorder, the sample size is still very small for making any generalizations especially given the very wide age range across patients in this study. Second, we do not have data about cognitive testing in these individuals, and the relationship with cognitive domains and behavior, sleep, and adaptive functioning could be very important. Third, parent questionnaires are effective for some things but are still limited tools. These questionnaires specifically have data supporting their use in populations with intellectual disability, but the sleep questionnaire, in particular, was still designed for typically developing children. This population would benefit from studies that do a more direct assessment of cognition and behavior with neuropsychological tests and sleep using actigraphy and/or polysomnography. Fourth, although the questionnaires used are standardized with published norms, the addition of control groups such as typically developing and children with a different genetic syndrome causing intellectual disability would have made for a stronger study.

Additional future directions should include more detailed neuropsychological assessments beyond just caregiver rating scales to identify any patterns of strengths and weaknesses. Adaptive and behavioral reporting utilized the respective instruments normative sample as a control group but our sleep reporting lacks such a control. Future directions could benefit from utilizing controls in sleep assessments. Sleep should be characterized in more detail using actigraphy and/or polysomnography. Detailed characterizations will help to prognosticate and guide families as well as serve as potential markers of response for any future disease-modifying therapies.

## 5. Conclusions

This study examined adaptive function, behavior, and sleep in a cohort of individuals with genetically confirmed KAT6A syndrome. Sleep was problematic in the majority of participants and adaptive function was uniformly low but problematic behaviors were not a big issue in this group. For researchers, this suggests that further studies to do detailed characterization of sleep and cognition in patients with KAT6A syndrome are crucial. In addition, it suggests that when designing outcome measures for a potential future clinical trial, that sleep and cognition, and function are potential targets but the behavior is not a major concern. For clinicians, this study underscores the need to take a detailed sleep history beyond just sleep-disordered breathing. Clinicians should also be aware that behavioral rates are low so if a child has significant behaviors to look harder for sleep-disordered breathing or environmental contributions. Lastly, clinicians should help families to ensure maximal educational and community supports for these patients because adaptive functioning is universally low even in patients with better overall cognition.

## Figures and Tables

**Table 1:** Adaptive and Behavioral Data.

	Late Truncating (*n* = 19)	Early Truncating (*n* = 3)	Missense/Splice Site (*n* = 4)
Age (years)	10.41 (range 3–30)	15 (range 4–33)	14.25 (range 3–35)
Sex	10/19 M (53%)	1/3 M (33%)	2/4 M (50%)
ABAS-3 ^a^	*n* = 17	*n* =3	*n* = 4
General Adaptive Composite	59.3 ± 12.3	58.3 ± 14.0	64.8 ± 16.5
Conceptual	60.9 ± 12.2	61.3 ± 13.6	61.5 ± 11.4
Social	59.7 ± 12.4	66.3 ± 17.4	68.8 ± 14.4
Practical	59.3 ± 11.7	60 ± 7	68.5 ± 17.6
CBCL/ABCL ^b^	*n* = 16	*n* = 2	*n* = 4
Total Problems ^c^	59.1 ± 9.3	47.5 ± 9.2	59 ± 8.4
Internalizing Problems ^c^	55.4 ± 8.8	49.5 ± 3.5	55.3 ± 11.2
Externalizing Problems ^c^	52.2 ± 10.2	45 ± 11.3	52.5 ± 8.0
Depressive Problems ^d^	60.3 ± 7.8	55.5 ± 6.4	64.5 ± 9.5
Anxious Problems ^d^	56 ± 5.6	50 ± 0	55.5 ± 8.4
Attention-Deficit/Hyperactivity Problems ^d^	61.8 ± 8.2	53.5 ± 4.9	57.5 ± 6.1

ABAS-3 Adaptive Behavior Assessment System, CBCL Child Behavior Checklist, ABCL Adult Behavior Checklist. ^a^ ABAS-3 scores are normalized such that mean = 100 and one standard deviation = 15; higher scores reflect better adaptive function. ^b^ Behavior checklist T scores indicate more adverse behaviors as the score increases. ^c^ Scores 60–63 in borderline clinical range and above 63 in the clinical range. ^d^ Scores 65–69 in borderline clinical range and above 69 in the clinical range. No differences between groups were statistically significant for any item.

**Table 2:** Sleep Data.

Sleep Problem	Late Truncating with Problem (%)	Early Truncating with Problem (%)	Missense/Splice Site with Problem	Total in Cohort with Problem (%)
Prolonged sleep initiation	8/18 (44%)	0/3 (0%)	1/4 (25%)	9/24 (36%)
Multiple or prolonged awakenings	10/18 (56%)	0/3 (0%)	3/4 (75%)	13/25 (52%)
Bruxism	10/18 (56%)	2/3 (67%)	2/4 (50%)	14/25 (56%)
Restless sleep	17/18 (94%)	1/3 (33%)	2/4 (50%)	20/25 (80%)
Snore	3/18 (17%)	1/3 (33%)	2/4 (50%)	6/25 (24%)
Apnea	0/18 (0%)	1/3 (33%)	2/4 (50%)	3/25 (12%)
Enuresis (7+ years)	11/13 (85%)	1/2 (50%)	1/3 (33%)	13/18 (72%)
Needing medication for sleep	13/18 (72%)	1/3 (33%)	2/4 (50%)	16/25 (64%)
Daytime drowsiness or somnolence (7+ years)	9/13 (69%)	1/2 (50%)	3/3 (100%)	13/18 (72%)
Sought advice or treatment for sleep	13/18 (72%)	1/3 (33%)	3/4 (75%)	17/25 (68%)
Feel they have a sleep problem	10/18 (56%)	1/3 (33%)	4/4 (100%)	15/25 (60%)

Sleep problems by variant-type. Significant rates of sleep maintenance issues, bruxism, restless sleep, enuresis, daytime drowsiness, and need for medication.

## Data Availability

The data presented in this study are available on request from the corresponding author. The data are not publicly available due to participant privacy.

## Supplementary Materials

The following are available online at https://www.mdpi.com/article/10.3390/brainsci11080966/s1, Table S1: Individual Participants and Variants.

## References

[1] Arboleda V.A., Lee H., Dorrani N., Zadeh N., Willis M., Macmurdo C.F., Manning M.A., Kwan A., Hudgins L., Barthelemy F. (2015). De novo nonsense mutations in KAT6A, a lysine acetyl-transferase gene, cause a syndrome including microcephaly and global developmental delay. Am. J. Hum. Genet..

[2] Tham E., Lindstrand A., Santani A., Malmgren H., Nesbitt A., Dubbs H.A., Zackai E.H., Parker M.J., Millan F., Rosenbaum K. (2015). Dominant mutations in KAT6A cause intellectual disability with recognizable syndromic features. Am. J. Hum. Genet..

[3] Millan F., Cho M.T., Retterer K., Monaghan K.G., Bai R., Vitazka P., Everman D.B., Smith B., Angle B., Roberts V. (2016). Whole exome sequencing reveals de novo pathogenic variants in KAT6A as a cause of a neurodevelopmental disorder. Am. J. Med. Genet. A.

[4] Kennedy J., Goudie D., Blair E., Chandler K., Joss S., McKay V., Green A., Armstrong R., Lees M., Kamien B. (2019). KAT6A Syndrome: Genotype-phenotype correlation in 76 patients with pathogenic KAT6A variants. Genet. Med..

[5] Thomas T., Voss A.K. (2007). The diverse biological roles of MYST histone acetyltransferase family proteins. Cell Cycle.

[6] Perez-Campo F.M., Costa G., Lie-A-Ling M., Stifani S., Kouskoff V., Lacaud G. (2014). MOZ-mediated repression of p16(INK) (4) (a) is critical for the self-renewal of neural and hematopoietic stem cells. Stem Cells.

[7] Fahrner J.A., Bjornsson H.T. (2019). Mendelian disorders of the epigenetic machinery: Postnatal malleability and therapeutic prospects. Hum. Mol. Genet..

[8] Alarcón J.M., Malleret G., Touzani K., Vronskaya S., Ishii S., Kandel E.R., Barco A. (2004). Chromatin acetylation, memory, and LTP are impaired in CBP+/- mice: A model for the cognitive deficit in Rubinstein-Taybi syndrome and its amelioration. Neuron.

[9] Korzus E., Rosenfeld M.G., Mayford M. (2004). CBP histone acetyltransferase activity is a critical component of memory consolidation. Neuron.

[10] Benjamin J.S., Pilarowski G.O., Carosso G.A., Zhang L., Huso D.L., Goff L.A., Vernon H.J., Hansen K.D., Bjornsson H.T. (2017). A ketogenic diet rescues hippocampal memory defects in a mouse model of Kabuki syndrome. Proc. Natl. Acad. Sci. USA.

[11] Achenbach T.M., Rescorla L. (2000). Manual for the ASEBA Preschool Forms & Profiles: An Integrated System of Multi-Informant Assessment.

[12] Achenbach T.M., Rescorla L. (2001). Manual for the ASEBA school-age forms & profiles: An integrated system of multi-informant Assessment.

[13] Achenbach T.M., Rescorla L. (2003). Manual for the ASEBA Adult Forms & Profiles: For Ages 18–59: Adult Self-Report, Adult Behavior Checklist.

[14] Harrison P.L., Oakland T., Psychological Corporation (2000). ABAS, Adaptive Behavior Assessment System: Manual.

[15] von Buttlar A.M., Zabel T.A., Pritchard A.E., Cannon A.D. (2021). Concordance of the Adaptive Behavior Assessment System, second and third editions. J. Intellect. Disabil. Res..

[16] Simonds J.F., Parraga H. (1982). Prevalence of sleep disorders and sleep behaviors in children and adolescents. J. Am. Acad. Child. Psychiatry.

[17] Simonds J.F., Parraga H. (1984). Sleep behaviors and disorders in children and adolescents evaluated at psychiatric clinics. J. Dev. Behav. Pediatr..

[18] Wiggs L., Stores G. (1996). Severe sleep disturbance and daytime challenging behaviour in children with severe learning disabilities. J. Intellect. Disabil. Res..

[19] Wiggs L., Stores G. (1998). Behavioural treatment for sleep problems in children with severe learning disabilities and challenging daytime behaviour: Effect on sleep patterns of mother and child. J. Sleep Res..

[20] Wiggs L., Stores G. (2004). Sleep patterns and sleep disorders in children with autistic spectrum disorders: Insights using parent report and actigraphy. Dev. Med. Child. Neurol..

[21] Johnson C.R., Turner K.S., Foldes E.L., Malow B.A., Wiggs L. (2012). Comparison of sleep questionnaires in the assessment of sleep disturbances in children with autism spectrum disorders. Sleep Med..

[22] Landrum M.J., Lee J.M., Benson M., Brown G.R., Chao C., Chitipiralla S., Gu B., Hart J., Hoffman D., Jang W. (2018). ClinVar: Improving access to variant interpretations and supporting evidence. Nucleic Acids Res..

[23] Schwarz J.M., Cooper D.N., Schuelke M., Seelow D. (2014). MutationTaster2: Mutation prediction for the deep-sequencing age. Nat. Methods.

[24] Stein M.A., Mendelsohn J., Obermeyer W.H., Amromin J., Benca R. (2001). Sleep and behavior problems in school-aged children. Pediatrics.

[25] Bornstein M.H., Hahn C.S., Suwalsky J.T. (2013). Developmental Pathways among Adaptive Functioning and Externalizing and Internalizing Behavioral Problems: Cascades from Childhood into Adolescence. Appl. Dev. Sci..

[26] Dekker M.C., Koot H.M., van der Ende J., Verhulst F.C. (2002). Emotional and behavioral problems in children and adolescents with and without intellectual disability. J. Child. Psychol. Psychiatry.

[27] Leader G., Forde J., Naughton K., Maher L., Arndt S., Mannion A. (2021). Relationships among gastrointestinal symptoms, sleep problems, challenging behaviour, comorbid psychopathology and autism spectrum disorder symptoms in children and adolescents with 15q duplication syndrome. J. Intellect. Disabil. Res..

[28] Fucà E., Costanzo F., Celestini L., Mandarino A., Vicari S. (2021). Characterization of Sleep Disturbances in Children and Adolescents with Down Syndrome and Their Relation with Cognitive and Behavioral Features. Int. J. Environ. Res. Public Health.

[29] Agar G., Brown C., Sutherland D., Coulborn S., Oliver C., Richards C. (2021). Sleep disorders in rare genetic syndromes: A meta-analysis of prevalence and profile. Mol. Autism.

