# Supplementary material for: Sleep, Behavior, and Adaptive Function in KAT6A Syndrome
Source: Brain Sci. 2021 Jul 23;11(8):966. doi: 10.3390/brainsci11080966 (PMC8393229; doi:10.3390/brainsci11080966)
Supplement: Supplementary file 1 [file brainsci-11-00966-s001.zip › brainsci-1241797-supplementary.pdf]

Table S1. Individual Participants and Variants.

Subject	Age	Sex	*KAT6A* Variant
1	10	M	c.5212 G>A; p.D1738N
2	3	F	c.4664G>A; p.S1555N
3	9	F	c.1862A>G; p.N621S°
4	4	F	c.1951_1954delCCTC; p.P651fsX47
5	8	M	c.693_705del; p.D232VfsX7
6	33	F	C.1136C>G; p.S379X
7	4	M	c.3429dupC; p.T1144HfsX31
8	13	M	c.3631_3632delGT; p.V1211X
9	18	F	c.3230delA; p.N1077MfsX46
10	8	F	c.4292dupT; pL1431FfsX8
11	3	F	c.4046_4047delAG p.E1349VfsX6
12	9	M	c.3830_3831insTT; p.R1278SfsX17
13	35	M	c.3040-1_3040delGA; p.K1014fs
14	10	F	c.4228_4232delAAAGA; p.K1410GfsX7
15	4	M	c.5645_5646delTTins10; p.V1882GfsX4
16	6	F	c.4089_4092dup; p.D1365KfsX3
17	5	M	c.3286_3287insC ;p.C1096SfsX6
18	7	F	c.4254_4257del; p.E1419WfsX12
19	18	M	c.3853_3862del10, p.Q1285RfsX6
20	30	M	c.3338C > G; p.S1113X
21	11	M	c.3385C>T; p.R1129X
22	8	F	c.3385C>T; p.R1129X
23	11	M	c.3385C>T; p.R1129X
24	12	F	c.4108G>T; pE1370X
25	11	M	c.3182T>G;p.L1061X
26	4	F	c.3070C>T; p.R1024X

° also has benign change c.4952C>T; p.P1651L
